# Effects of Trimethylamine N-Oxide in Improving Exercise Performance in Mice: A ^1^H-NMR-Based Metabolomic Analysis Approach

**DOI:** 10.3390/molecules29174128

**Published:** 2024-08-30

**Authors:** Hong Zou, Lijing Gong, Zhiyuan Wang, Caihua Huang, Yue Luo, Xiao Jia, Jingjing Yu, Donghai Lin, Yimin Zhang

**Affiliations:** 1 Key Laboratory of Ministry of Education of Exercise and Physical Fitness, Beijing Sport University, Beijing 100084, China; zou_hong0402@126.com (H.Z.); lijing.gong@bsu.edu.cn (L.G.); zhiyuanw2024@163.com (Z.W.); jiaxiao@bsu.edu.cn (X.J.); yujingjing@bsu.edu.cn (J.Y.); 2 Physical Education Department, Xiamen University, Xiamen 361005, China; 3 China Institute of Sports and Health, Beijing Sport University, Beijing 100084, China; 4 Research and Communication Center of Exercise and Health, Xiamen University of Technology, Xiamen 361021, China; caihua.huang@foxmail.com; 5 School of Physical Education and Health, Chongqing College of International Business and Economics, Chongqing 401520, China; luoyue980803@163.com; 6 Key Laboratory for Chemical Biology of Fujian Province, MOE Key Laboratory of Spectrochemical Analysis & Instrumentation, College of Chemistry and Chemical Engineering, Xiamen University, Xiamen 361005, China

**Keywords:** TMAO, NMR-based metabolomics, exercise, skeletal muscle

## Abstract

To improve exercise performance, the supplement of nutrients has become a common practice before prolonged exercise. Trimethylamine N-oxide (TMAO) has been shown to ameliorate oxidative stress damage, which may be beneficial in improving exercise capacity. Here, we assessed the effects of TMAO on mice with exhaustive swimming, analyzed the metabolic changes, and identified significantly altered metabolic pathways of skeletal muscle using a nuclear magnetic resonance-based (NMR-based) metabolomics approach to uncover the effects of TMAO improving exercise performance of mice. We found that TMAO pre-administration markedly prolonged the exhaustive time in mice. Further investigation showed that TMAO pre-administration increased levels of 3-hydroxybutyrate, isocitrate, anserine, TMA, taurine, glycine, and glutathione and disturbed the three metabolic pathways related to oxidative stress and protein synthesis in skeletal muscle. Our results provide a metabolic mechanistic understanding of the effects of TMAO supplements on the exercise performance of skeletal muscle in mice. This work may be beneficial in exploring the potential of TMAO to be applied in nutritional supplementation to improve exercise performance. This work will lay a scientific foundation and be beneficial to exploring the potential of TMAO to apply in nutritional supplementation.

## 1. Introduction

As the largest metabolic tissue in the human body, skeletal muscle counts for approximately 40% of the total body weight and coordinates and supports the body’s movement with other tissues such as bones, nerves, and blood vessels [1,2]. Skeletal muscle usually generates reactive oxygen species (ROS) in conditions of both rest and contraction due to its rich mitochondria content [3]. Many studies have confirmed that prolonged or high-intensity exercise can induce the production of a large amount of ROS in skeletal muscles and blood, leading to oxidative damage and, subsequently, functional disorders [4,5]. Therefore, it’s particularly important to exclude the excessive ROS and maintain the strength and function of skeletal muscle. Although the impact of exogenous nutritional supplements on the body’s redox homeostasis and physical performance is still uncertain [6], many studies have confirmed that various nutritional supplementation can alleviate oxidative stress damage in tissues and improve exercise performance [7,8,9].

Trimethylamine N-oxide (TMAO) is primarily present in animal tissues, with concentrations notably higher in marine animals compared to other organisms [10], and in marine animals, the content of TMAO increases with the increase of habitat depth. As an endogenous compound, TMAO gives marine organisms a distinctive and appealing flavor and is a characteristic substance that distinguishes marine organisms from other animals [11]. Recently, TMAO has been found to alleviate the negative effects of oxidative stress in MC65 human neuroblastoma cells [12] and diabetic rats [13] and has attracted increasing interest from scientists. Our previous work has also confirmed that TMAO treatment can partially alleviate the H_2_O_2_-induced oxidative stress damage in vitro [14]. Therefore, we speculate that TMAO may have the potential to prolong the exercise duration and enhance the exercise performance of mice.

Metabolomics, a powerful analytical approach, is often applied to explore metabolic mechanisms of evaluating the impact of food and nutritional supplements on the body [15,16,17]. Alternations in metabolites that are downstream products of gene transcription can be used to visualize changes in overall metabolism. As one of the most widely used detection techniques, nuclear magnetic resonance (NMR) plays an important role in metabolomics research because of the simple sample pretreatment, quick detection, and objective and quantitative measurements [18]. Previously, we performed NMR-based metabolomic analyses to explore both the effects of α-Ketoglutarate supplementation on C2C12 myoblasts in different energy states [19] and the effects of lactate treatment on disuse-induced muscle atrophy [20].

Here, we studied the effects of TMAO administration on endurance time in a forced swimming test with mice, performed NMR-based metabolomic analyses of skeletal muscle, and tried to reveal the metabolic mechanism behind the potential benefits of TMAO. This study could significantly advance our understanding of the therapeutic potential of TMAO in regulating metabolic changes and improving muscle exercise performance. The findings may also contribute valuable insights for the strategic application of TMAO supplementation in sports fields.

## 2. Results

### 2.1. Effect of TMAO on the Swimming Exhaustive Time

**Figure 1:**
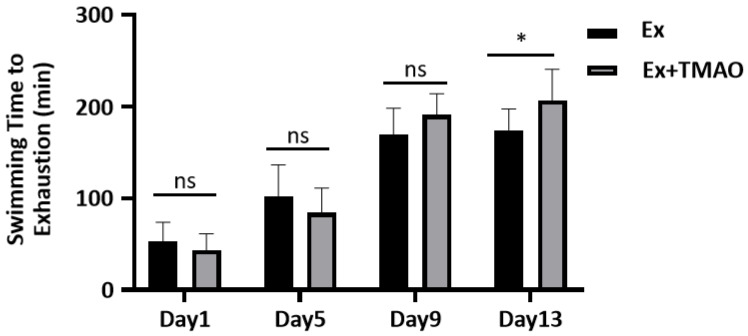
The comparison of swimming exhaustive time of mice between the Ex and Ex + TMAO groups. Statistical significances: ns, *p* > 0.05, * *p* < 0.05.

To evaluate the effect of TMAO on improving exercise performance, we conducted a comparative analysis of the swimming exhaustive time of mice in the swim-exhausted control (Ex) group and swim-exhausted + TMAO-supplemented (Ex + TMAO) group separately on day 1, 5, 9 and 13. We found that the exhaustive time of mice in the Ex + TMAO group on day 13 was significantly longer than that of the Ex group (*p* < 0.05, Figure 1). The result indicates that TMAO-administration can significantly improve the exercise performance of mice.

### 2.2. NMR Spectra of Hydrophilic Metabolites Extracted from Skeletal Muscle

**Figure 2:**
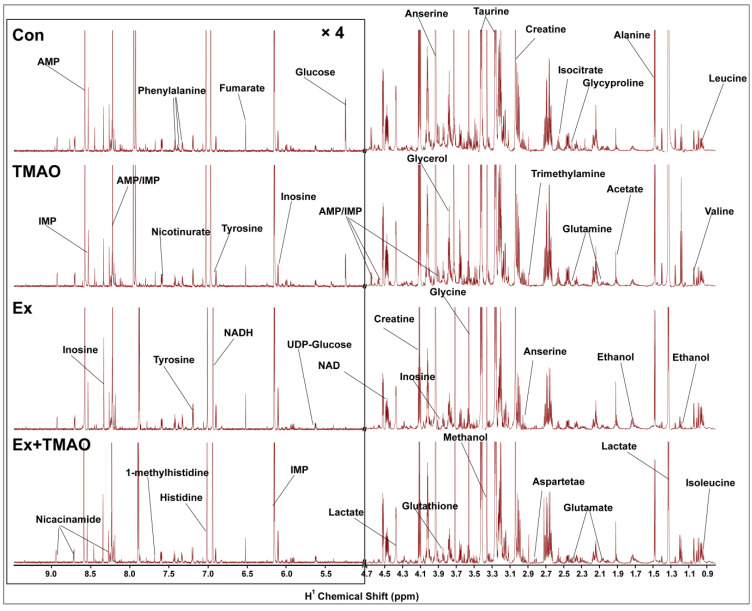
Typical 850 MHz ^1^H-NMR spectra recorded on aqueous extracts derived from the skeletal muscle of mice in four groups.

The typical 850 MHz ^1^H−NMR spectra of assigned hydrophilic metabolites extracted from the skeletal muscle of mice in the control (Con), TMAO-supplemented (TMAO), Ex, and Ex + TMAO groups were shown (Figure 2). By comparing these spectra intuitively, it was evident that the positions of spectral peaks were generally the same among these four groups, but the differences among each group were significant in the intensity of individual peaks. Based on the NMR spectra, we successfully identified and confirmed all 37 metabolites (Table S1) with the aid of 2D ^1^H-^13^C HSQC spectra (Figure S1).

### 2.3. Multivariate Statistical Analysis for NMR Spectra of Metabolites Extracted from Skeletal Muscle

**Figure 3:**
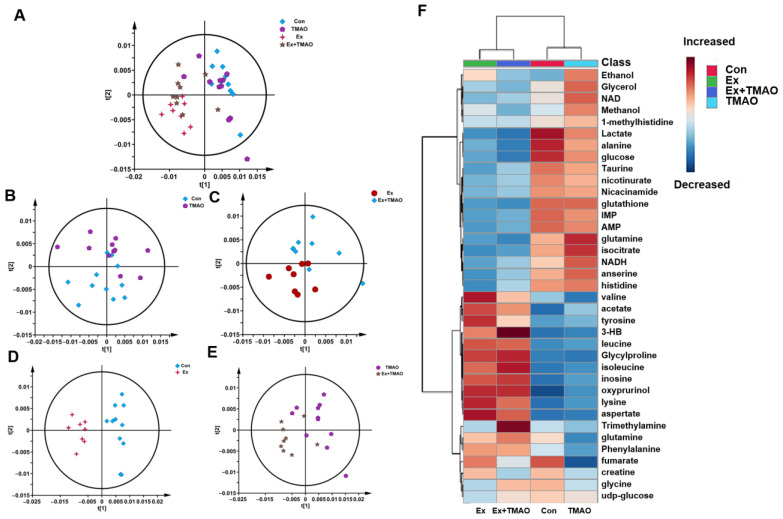
Multivariate statistical analysis for NMR spectra of metabolites extracted from skeletal muscle. (**A**) PCA scores plot of four groups. (**B**) PCA scores plot of the Con group vs. the TMAO group. (**C**) PCA scores plot of the Ex group vs. the Ex + TMAO group. (**D**) PCA scores plot of the Con group vs. the Ex group. (**E**) PCA scores plot of the TMAO group vs. the Ex + TMAO group. (**F**) Hierarchical cluster analysis of four groups.

To comprehensively understand the metabolic characteristics of skeletal muscles in mice, a multivariate data analysis was conducted based on NMR spectra. Firstly, the unsupervised PCA model was carried out to distinguish the metabolic patterns of Con vs. TMAO, Ex vs. Ex + TMAO, Con vs. Ex, and TMAO vs. Ex + TMAO (Figure 3A). The results showed that the metabolic profile of the Con group was close to the TMAO group (Figure 3B), while the Ex group was distinctly separated from the Ex + TMAO group (Figure 3C). In addition, the metabolic profiles of the Con vs. Ex, TMAO vs. Ex + TMAO have a certain extent of differentiation (Figure 3D,E). The distinguishing characteristics of the PCA model were further verified by the hierarchical cluster analysis. Results showed obvious differences between the Con, TMAO groups and the Ex, Ex + TMAO groups, while there were also significant differences between the Con group vs. the Ex group, the TMAO group vs. the Ex + TMAO group, and slightly different clusters of the Ex group vs. the Ex + TMAO group (Figure 3F), which were accordance with the PCA scores plot.

**Figure 4:**
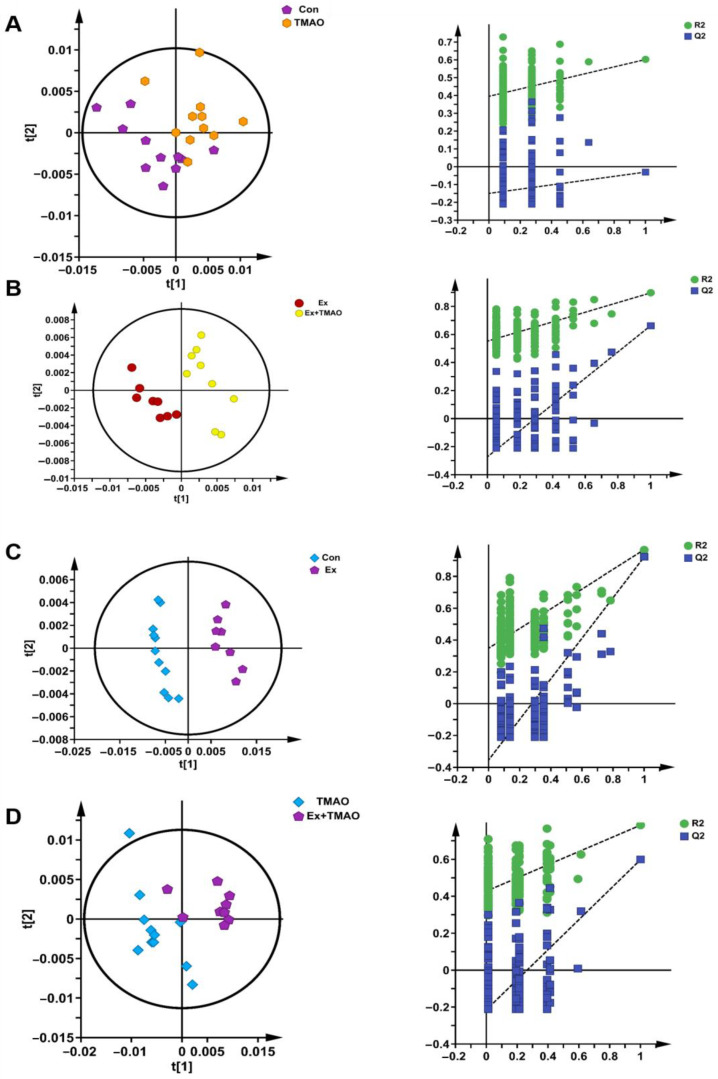
The score plots and cross-validation plots of PLS−DA models of ^1^H−NMR spectra recorded on aqueous extracts derived from the skeletal muscle of mice. (**A**) The Con group vs. the TMAO group. (**B**) The Ex group vs. the Ex + TMAO group. (**C**) The Con group vs. the Ex group. (**D**) The TMAO group vs. the Ex + TMAO group.

Additionally, we also established a supervised PLS−DA model to screen the differences in metabolic patterns among groups more easily. The PLS−DA scores plots displayed the close metabolic pattern of the Con group and TMAO groups (Figure 4A). In addition, the other PLS−DA score plots demonstrated clear separations between the Ex and Ex + TMAO groups, the Con and Ex groups, and the TMAO and Ex + TMAO groups, and the cross-validation plots also displayed reliably from the obtained R^2^ and Q^2^ values of the three PLS−DA, as follows: R^2^ = 0.801 and Q^2^ = 0.497 for the Ex vs. Ex + TMAO groups; R^2^ = 0.921 and Q^2^ = 0.967 for the Con vs. Ex groups; R^2^ = 0.784 and Q^2^ = 0.598 for the TMAO vs. Ex + TMAO groups (Figure 4B–D).

### 2.4. Identifications of Differential Metabolites of Aqueous Extracts Derived from Skeletal Muscle of Mice

To quantitatively compare the concentrations of metabolites, we calculated the relative integrations of metabolites after fully normalizing the integration of 1D ^1^H−NMR spectra of aqueous metabolites. Using the criterion of *p* < 0.05, the differential metabolites were shown between the four groups (Table S2). In total, for the Ex + TMAO group vs. the Ex group, 15 differential metabolites were identified, including 8 downregulated metabolites and 7 upregulated metabolites; for the Ex vs. Con groups, 31 differential metabolites were identified, including 20 downregulated metabolites and 11 upregulated metabolites; for the Ex + TMAO vs. TMAO groups, 34 differential metabolites were identified, including 21 downregulated metabolites and 13 upregulated metabolites.

### 2.5. Identifications of Important Metabolites of Aqueous Extracts Derived from Skeletal Muscle of Mice

**Figure 5:**
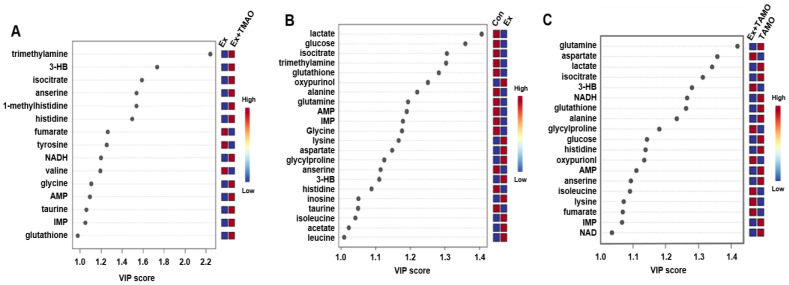
VIP of metabolites screened by the PLS-DA model of skeletal muscle of mice. (**A**) The important metabolites of the Ex group vs. the Ex + TMAO group. (**B**) The important metabolites of the Con group vs. the Ex group. (**C**) The important metabolites of the TMAO group vs. the Ex + TMAO group.

From the established PLS−DA models above, we screened important metabolites that had significant differential contributions to the two metabolic modes using VIP as the screening criteria (VIP ≥ 1). The results are shown in Figure 5. For the Ex + TMAO vs. Ex groups, 15 important metabolites were screened, including 3 downregulated metabolites and 12 upregulated metabolites; for the Ex vs. Con groups, 22 differential metabolites were identified, including 13 downregulated metabolites and 9 upregulated metabolites; for the Ex + TMAO vs. TMAO groups, 19 differential metabolites were identified, including 12 downregulated metabolites and 7 upregulated metabolites.

### 2.6. Identifications of Characteristic Metabolites of Aqueous Extracts Derived from Skeletal Muscle of Mice

The characteristic metabolites were screened using the criteria VIP ≥ 1 and *p* < 0.05. The results are shown in Table 1. For the Ex + TMAO vs. Ex, 10 characteristic metabolites were screened, including valine, fumarate, tyrosine, 3HB, isocitrate, anserine, TMA, taurine, glycine, and glutathione. For the Ex vs. Con, 21 characteristic metabolites were identified, including leucine, alanine, glutamine, isocitrate, anserine, taurine, glycine, glutathione, lactate, glucose, IMP, Histidine, AMP, isoleucine, 3HB, acetate, lysine, glycylproline, aspartate, oxypurinol, and inosine; For the Ex + TMAO vs. TMAO, 19 characteristic metabolites were identified, including alanine, glutamine, isocitrate, anserine, glutathione, lactate, NAD, glucose, IMP, NADH, histidine, AMP, isoleucine, 3HB, lysine, glycylproline, fumarate, oxypurinol, and aspartate.

### 2.7. Identifications of Significantly Altered Metabolic Pathways of Aqueous Extracts Derived from Skeletal Muscle of Mice

**Figure 6:**
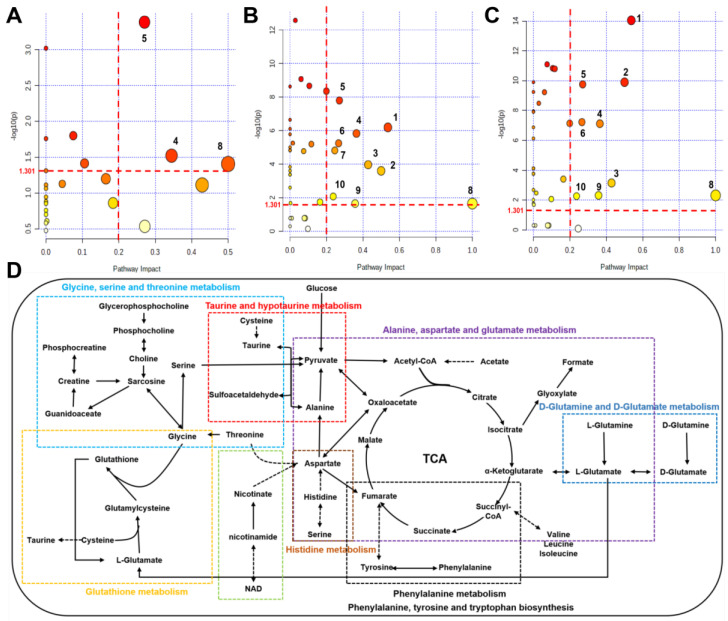
Metabolic pathways and metabolic pathway schematic diagram were significantly altered in skeletal muscles of mice based on the KEGG database. (**A**) Ex + TMAO vs. Ex; (**B**) Ex vs. Con; (**C**) Ex + TMAO vs. TMAO; (**D**) metabolic pathway schematic diagram. (1) alanine, aspartate and glutamate metabolism; (2) D-Glutamine and D-glutamate metabolism; (3) taurine and hypotaurine metabolism; (4) glutathione metabolism; (5) histidine metabolism; (6) nicotinate and nicotinamide metabolism; (7) glycine, serine and threonine metabolism; (8) phenylalanine, tyrosine and tryptophan biosynthesis; (9) phenylalanine metabolism; (10) glycerolipid metabolism.

Using the pathway analysis module on MetaboAnalyst 5.0, the metabolic pathways that were significantly altered were screened with a criterion of PIV > 0.2 [equivalently corresponding: −log10(p) > 1.301] and *p* < 0.05. We identified 10 metabolic pathways: (1) alanine, aspartate, and glutamate metabolism; (2) D-glutamine and D-glutamate metabolism; (3) taurine and hypotaurine metabolism; (4) glutathione metabolism; (5) histidine metabolism; (6) nicotinate and nicotinamide metabolism; (7) glycine, serine and threonine metabolism; (8) phenylalanine, tyrosine and tryptophan biosynthesis; (9) phenylalanine metabolism; (10) glycerolipid metabolism (Figure 6A–C and Table S3). The comparison of Ex + TMAO vs. Ex identified 3 significant metabolic pathways, the comparison of Ex vs. Con identified all 10 significant metabolic pathways, and the comparison of Ex + TMAO vs. TMAO identified 9 significant metabolic pathways.

To systematically and intuitively display the metabolic regulation of TMAO on skeletal muscles of mice with exhausted swimming training, we drew a metabolic pathway schematic diagram on the foundation of metabolic pathway analysis on Kyoto Encyclopedia of Genes and Genomes (KEGG) database (Figure 6D) and MetaboAnalyst 5.0. From the metabolic pathway diagram, it can be found that the metabolic pathways affected in the skeletal muscles of exercise mice by TMAO administration mainly manifested in oxidative stress, amino acid metabolism, and other aspects.

## 3. Discussion

It is well known that skeletal muscle generates strength through the contraction of muscle fibers during physical activity and strictly regulates this process through stress responses and antioxidant defense systems [21,22]. During strenuous exercise, the contraction of skeletal muscle is usually accompanied by oxidative stress out of balance. If peroxide products are not cleared in a timely manner, they may disrupt the normal function of skeletal muscle [23]. Antioxidant supplementation is considered to be beneficial at this time.

In this research, we exploited the effect of 13-day TMAO administration on continuous swimming to exhaustion time in mice and addressed the unclassified metabolic mechanisms using NMR-based metabolomic analyses. Our study demonstrated that TMAO supplementation increased the duration of continuous swimming until exhaustion. Furthermore, the metabolomic analysis identified significant alterations in 9 metabolic pathways, including alanine, aspartate, and glutamate metabolism; D-glutamine and D-glutamate metabolism; taurine and hypotaurine metabolism; glutathione metabolism; histidine metabolism; nicotinate and nicotinamide metabolism; phenylalanine, tyrosine and tryptophan biosynthesis; phenylalanine metabolism; glycerolipid metabolism.

The analysis of metabolic pathways showed that glutathione metabolism pathways are significantly disturbed after TMAO administration, which is a key metabolic pathway closely related to oxidative stress. Furthermore, our data also showed seven characteristic metabolites were significantly elevated, including 3HB, isocitrate, anserine, TMA, taurine, glycine, and glutathione (Table 1). Glutathione and taurine are closely related to oxidative stress. Glutathione can protect against the damage caused by attacks on cells by free radicals, lipid peroxides, and other substances closely related to oxidative stress and is considered a common and important antioxidant in animals and plants [24,25]. Glutathione generally does not usually act alone but is only involved in metabolic and biochemical reactions such as synthesizing protein, transporting amino acids, and regulating the activity of antioxidant enzymes [26]. Taurine is considered a natural antioxidant that can contribute to scavenging oxidative stress products caused by diseases, strenuous exercise, high temperatures, and so on [27]. Both metabolites contribute to improving the body’s antioxidant capacity. In addition, isocitrate has been proven to protect DJ-1 deficient dopaminergic cells from oxidative stress damage via isocitrate dehydrogenase (IDH) [28]. Glycine is the main amino acid in the human body and has been proven to participate in the body’s antioxidant response by altering the glutathione metabolism pathway [29]. Other studies have suggested that the addition of glycine can effectively enhance the antioxidant capacity of mice embryos [30]; at the same time, glycine intervention has also been proven to exert a protective effect on the myocardium of diabetes rats by improving oxidative stress [31]. The results of this study showed that the concentrations of isocitrate, glycine, taurine, and glutathione were all down-regulated in mice after exhaustive swimming, while the concentrations of these metabolites were upregulated after TMAO intervention, indicating that TMAO can enhance exercise performance via activating glutathione metabolism pathways.

Although, the impact of TMAO on human health is controversial [32]. However, there is growing evidence that TMAO is likely to be a marker rather than a cause of disease, and its protective effects have been demonstrated in a number of animal experiments [33]. The role of TMAO in alleviating oxidative stress has also been confirmed [12,13], as well as in our previous research [14]. As already reported, TMAO’s precursors, L-carnitine supplementation (2–3 g/d) for 3 consecutive weeks can effectively alleviate pain [34,35,36], L-carnitine supplementation for 9 weeks combined with resistance training resulted in a significant increase in total antioxidant capacity and glutathione peroxidase activity, as well as a decrease in malondialdehyde concentration [37]. This present study demonstrated that TMAO supplementation prolonged the duration of exhaustive swimming in mice, which may be related to the enhancement of total antioxidant capacity in mice. 

Furthermore, some studies showed that when TMAO or its precursors are supplied at a lower recommended dose, the protective effects on atherosclerosis, hypertension, and cholesterol [38,39,40]. Studies reported a significant decrease in urinary TMAO levels after acute exercise in elite female athletes and women and men with exercise habits [41,42]. A study showed that TMAO levels dropped by about 20–21% after 1 h of exercise and then slowly returned to baseline levels [43]. Many acute exercise experiments, including mice and elite athletes, have also shown changes in TMAO levels in vivo before and after exercise [44,45,46]. Therefore, TMAO may play a role in exercise, but the specific effects and mechanisms are still unclear. In this study, 9-week-old C57BL/6J male mice were orally administered TMAO for approximately 13 days and tested every 4 days with an exhaustive swimming test. The results showed that there was a significant extension in the last bout of duration of continuous exercise after the TMAO supplement, indicating that TMAO administration could prolong the duration of swimming exhaustion of mice and enhance their aerobic capacity. This study is the first to apply TMAO as an administration to enhance the aerobic capacity of skeletal muscles in mice.

In addition, our study identified that histidine metabolism, phenylalanine, tyrosine, and tryptophan biosynthesis were significantly disturbed metabolic pathways after TMAO administration. Upregulated levels of glycine were observed in the Ex + TMAO group when compared to the Ex group. These metabolites were all related to amino acid metabolism. Some studies showed that TMAO in deep-sea fish could protect proteins from the harmful effects of urea [47,48,49]. Thus, TMAO supplementation to prolong the swimming exhaustion time of mice may also be related to the function of TMAO in maintaining protein stability, which needs to be investigated in future studies.

In our experiment, we used a similar method but modified it by reducing the tethered weight from 10% to 5% of the mouse’s body weight, as done by Chen et al. [50]. We also implemented all standard precautionary measures, but unfortunately, some mice died due to asphyxiation, while others perished from drowning. We acknowledge that we cannot definitively state that no other training regimen could have addressed our research question. Similar to the choice of running, which cannot be proven to be the only viable exercise option, we chose swimming because of its beneficial aspects and our interest in exploring whether TMAO supplementation could improve performance in this activity. We deeply regret the resulting mortality and are committed to ensuring the highest ethical standards in our research. In future studies, we will seriously consider alternative approaches to fatigue testing, such as treadmill running or voluntary running in an exercise wheel, to minimize harm to the animals. In addition, we will explore exhaustive running and hanging tests as potential alternatives. Some international funding agencies have started to ban forced swim tests. We support this viewpoint to improve the welfare of the test animals.

## 4. Materials and Methods

### 4.1. Animals and Ethics Statement

Sixty C57BL/6J male mice (about 11 weeks) were purchased from Beijing Huafukang Biotechnology Co., Ltd. (Beijing, China). All mice were randomly divided into a sedentary group (*n* = 30) and an exhaustive swimming group (*n* = 30). The sedentary mice were randomly grouped as control mice (Con, *n* = 15) and TMAO-supplemented mice (TMAO, *n* = 15). The swim-exhausted mice were randomly divided into two groups: swim-exhausted control mice (Ex, *n* = 15) and swim-exhausted + TMAO-supplemented mice (Ex + TMAO, *n* = 15). The mice underwent 1 week of environmental adaptation before the start of the study. The study was approved by the Sports Science Experiment Ethics Committee of Beijing Sports University (2021127A) while strictly following the “Instructive Notions with Respect to Caring for Laboratory Animals” issued by the Ministry of Science and Technology of the People’s Republic of China. All mice were maintained in the Experimental Animal Center of Beijing Sports University (Beijing, China) under controlled environmental conditions (12:12 h light-dark cycle) with 22–24 °C temperature, 55–70% relative humidity, and fed rodent chow and water ad libitum.

### 4.2. Swimming Exhaustive Test Protocol

Mice in the Ex and Ex + TMAO groups were acclimated to the swimming training for two weeks (5 times per week) before the exhaustive tests. Exhaustive swimming was performed in a plastic water tank (100 cm × 60 cm × 40 cm) with a water temperature of 30 ± 2 °C. The adaptive swimming training plan (Table S4) included zero load in the first week and weight-bearing in the second week of adaptive swimming training.

**Figure 7:**
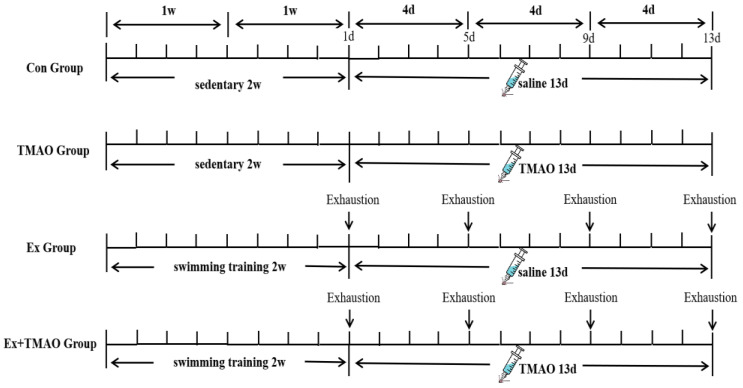
Workflow of the exhaustive swimming experiment.

Then, mice in the Ex and Ex + TMAO groups performed four exhaustive swimming bouts with a 4-day interval between each bout. During the exhaustive swimming test, the mice bore a 5% burden of their own body weight. Before each test, the mice were weighed to adjust their load weight, which was carried by wrapping a lead skin around the tail. The specific schematic diagram of the experimental design is shown in Figure 7. During swimming, mice were constantly driven away to avoid floating. The duration time was recorded until exhaustion from swimming. The standard of exhaustion was that the head of mice couldn’t protrude from the water surface for more than 10 s [50,51]. 

### 4.3. TMAO Administration

TMAO (Sigma-Aldrich, St. Louis, MO, USA, 95%, 317594-5G) was fully diluted in normal saline (NS) and administered intragastrically (800 mg/kg body weight) daily for thirteen days. Mice in TMAO and Ex + TMAO groups were given i.g. TMAO daily, while Con and Ex groups were given i.g. with NS at the same time. 

### 4.4. Samples Collection

All the mice were sacrificed after the last bout of swimming by cervical dislocation. The gastrocnemius of the mice was immediately removed and pre-cooled with liquid nitrogen before being stored at −80 °C.

### 4.5. Extraction of Mice Skeletal Muscle’s Aqueous Metabolites

For NMR-based metabolomic analysis, aqueous metabolites of the skeletal muscle of mice were extracted as in previous work [20]. After being taken out of the −80 °C ultra-low temperature refrigerator, the gastrocnemius was thawed at 4 °C, the surface fascia and the Achilles tendon of gastrocnemius was removed, and approximately 70 mg of the gastrocnemius was cut and put into 2 mL homogenizer tube. Then the pre-cooled methanol (4 mL/g) and two small magnetic beads were added into the tube and homogenized under the parameter of 65 Hz for 60 s. A mix of pre-cooled chloroform and ultrapure water (4: 2.854 mL/g) was added to the homogenizer tube and underwent the same protocol including homogenizing (65 Hz, 60 s), vortexing (2 min), and centrifuging (12,000 rpm, 15 min at 4 °C). Subsequently, the aqueous extracts in the upper layer were transferred to a new EP tube, the methanol volatized clean by a nitrogen blowing instrument, and the remaining aqueous solution was freeze-dried by lyophilizer.

### 4.6. Preparation for NMR Sample

The freeze-dried samples were suspended into 550 µL NMR buffer (D_2_O with 50 mM PBS, pH 7.4, 1 mM TSP), vortexed (30 s), and centrifuged (2000 rpm, 5 min, 4 °C). Then the sample was transferred to 5 mm NMR tube and centrifuged (1000 rpm, 5 min) using nuclear magnetic tube low-speed centrifuge.

### 4.7. Measurements of Nuclear Magnetic Resonance Spectroscopy

All NMR spectroscopy was acquired at 298 K on a Bruker AVANCE III 850 MHz NMR spectrometer equipped with a TCI cryoprobe (Bruker Bio Spin, Rheinstetten, Germany). One-dimensional (1D) ^1^H-NMR spectra of skeletal muscle sample were acquired using the NOESYGPPR1D pulse sequence (RD-G1-90°-tl-90°-τm-G2-90°-ACQ). Pulsed gradients (G1, G2) were used to improve water quality; short delay (t1):4 μs; mixing time (τm): 10 ms; spectral width: 20 ppm; acquisition time (ACQ): 1.93 s; number of scans (NS): 128; number of time domain data points (TD): 64 K.

Resonances of aqueous metabolites of the skeletal muscle were assigned based on the 1D ^1^H-NMR spectra by a combination of Chenomx NMR Suite software (version 8.3; Chenomx Inc., Edmonton, AB, Canada), the Human Metabolome Database (HMDB; http://www.hmdb.ca/, accessed on 3 December 2022), and relevant published references [14,20]. All resonances of the identified aqueous metabolites were confirmed via 2D ^1^H-^13^C HSQC spectra (detailed information showed as Supplemental Materials). 

### 4.8. Preprocess and Analysis of NMR Spectra

Preprocess of all 1D ^1^H-NMR spectra, including phase correction, baseline correction, and TSP calibration, were performed by MestReNova software (Version 9.0, Mestrelab Research S.L., La Coruña, Spain). All 1D ^1^H−NMR spectra were superimposed, and the stacked spectral peaks were aligned. The spectra region of δ 5.0–4.8 was excluded to eliminate confusion caused by water peaks, then the residue region of δ 9.5–0.8 was segmented with a width of 0.001 ppm for binning. To increase clarity, the area between 5.0 and 9.1 ppm has been magnified 10 times compared to the area between 0.8 and 4.8 ppm. Integration of each peak was normalized through the TSP spectral integral value using Matlab R2014b software to indicate the relative level of metabolites. The 2D ^1^H-^13^C HSQC spectra were preprocessed using TopSpin software (Version 3.5 pl 5, Bruker Bio Spin, Rheinstetten, Germany).

SIMCA-p (version 14.1.0, MKS Umetrics, Umea AB, Sweden) was carried out to perform multivariate statistical analysis based on a normalized data matrix. Firstly, the principal component analysis (PCA) was established to show the metabolic profiles among the groups and between samples. Then, on the basis of observing the differentiation trend of the above metabolic patterns, partial least-squares discriminant analysis (PLS−DA) was used to classify the grouping trend. The robustness of the established PLS-DA model was evaluated using R^2^ and Q^2^, which were usually calculated by a 200-times permutation test. The closer the values of R^2^ and Q^2^ to 1, the higher the reliability of the PLS−DA model. According to the PLS−DA model, metabolites with variable importance in projection (VIP) score > 1 were considered significant metabolites.

### 4.9. Metabolic Pathway Analysis

Metabolic pathway analysis was performed by MetaboAnalyst 5.0 (https://www.metaboanalyst.ca, accessed on 10 February 2023) to identify significantly altered metabolic pathways among groups. A combination of metabolite enrichment analysis and pathway topology analysis (PTA) were applied to determine these metabolic pathways. The metabolite enrichment analysis was evaluated using statistically significant *p*-values, with *p* < 0.05, to identify significant differential metabolites between groups. Moreover, path impact value (PIV), the other parameter for screening significantly altered metabolic pathways, was calculated by PTA. Finally, metabolic pathways that meet the needs of *p* < 0.05 and PIV > 0.2 were defined as characteristic metabolic pathways that can significantly alter metabolic patterns. 

### 4.10. Statistical Analysis

In MatLab R2014b software, the relative concentrations of metabolites were shown by relative integral values. Based on the integral values of metabolites, One-way ANOVA followed by Tukey’s multiple comparisons tests was used to quantitatively compare the relative levels of designated metabolites among four groups with GraphPad Prism software (version 8.3.0, La Jolla, CA, USA). All data were presented as the mean ± standard deviation (SD). The statistical significance was identified with *p*-values as follows: NS: *p* > 0.05, *: *p* < 0.05, **: *p* < 0.01, ***: *p* < 0.001, and ****: *p* < 0.0001.

## 5. Conclusions

In summary, in this study, we designed an exhaustive swimming experiment in mice, and on the basis of confirming that TMAO administration prolonged the duration of swimming exhaustion, we also performed an NMR-based metabolomic analysis of skeletal muscles in mice. Our findings revealed that the metabolic profiles of the skeletal muscles of mice were distinctly different with or without TMAO pre-administration prior to strenuous exercise, and further analyses suggested that TMAO may play an important role in oxidative stress and protein anabolism during intense exercise. Additionally, our findings suggested that TMAO administration didn’t alter the metabolic patterns of mice under quiet conditions, whereas prior to strenuous exercise, TMAO administration resulted in an increase the levels of the following metabolites: 3HB, isocitrate, anserine, TMA, taurine, glycine, glutathione, and caused perturbations in the following three metabolic pathways: glutathione metabolism, histidine metabolism, phenylalanine, tyrosine, and tryptophan biosynthesis. This study lays the scientific foundation of and is beneficial to exploring the potential of TMAO in enhancing the aerobic capacity of skeletal muscles in mice. However, the specific mechanism of TMAO in improving aerobic capacity should be continuously conducted in the future.

## Figures and Tables

**Table 1:** Characteristic metabolites in skeletal muscle of mice between groups.

Metabolites	Multiple Comparisons		
	Ex + TMAO vs. Ex	Ex vs. Con	Ex + TMAO vs. TMAO
Leucine	ns	↓	ns
Isoleucine	ns	↑	↑
Valine	↓	ns	ns
Ethanol	ns	ns	ns
3HB	↑	↑	↑
Alanine	ns	↓	↓
Lysine	ns	↑	↑
Acetate	ns	↑	ns
Glycylproline	ns	↑	↑
Glutamate	ns	ns	ns
Glutamine	ns	↓	↓
Isocitrate	↑	↓	↓
Anserine	↑	↓	↓
Aspartate	ns	↑	↑
TMA	↑	ns	ns
Taurine	↑	↓	ns
Glycine	↑	↓	ns
Glutathione	↑	↓	↓
Lactate	ns	↓	↓
NAD	ns	ns	↓
Glucose	ns	↓	↓
IMP	ns	↓	↓
Fumarate	↓	ns	↑
NADH	ns	ns	↓
Histidine	ns	↓	↓
Tyrosine	↓	ns	ns
Oxypurinol	ns	↑	↑
Inosine	ns	↑	ns
AMP	ns	↓	↓

Note: ↓: downregulated; ↑: upregulated. Statistical significances: ns, *p* > 0.05.

## Data Availability

The data is contained within the article or Supplementary Materials.

## Supplementary Materials

The following supporting information can be downloaded at https://www.mdpi.com/article/10.3390/molecules29174128/s1, **Table S1**: Identified metabolites in ^1^H NMR spectra of aqueous extracts derived from the four groups of skeletal muscle of mice; Table S2. Multiple comparisons of relative integration of aqueous metabolites from skeletal muscle of mice; Figure S1: Typical 2D ^1^H-^13^C HSQC spectrum of aqueous extracts derived from the four groups of mouse skeletal muscles recorded on 850 MHz NMR spectrometer; Table S3: Metabolic pathways were significantly altered in skeletal muscles of four groups of mice; Table S4: The design of 13-day adaptive swimming training.
