# Supplementary material for: Effects of Trimethylamine N-Oxide in Improving Exercise Performance in Mice: A 1H-NMR-Based Metabolomic Analysis Approach
Source: Molecules. 2024 Aug 30;29(17):4128. doi: 10.3390/molecules29174128 (PMC11397221; doi:10.3390/molecules29174128)
Supplement: Supplementary file 1 [file molecules-29-04128-s001.zip › molecules-3137046-supplementary.pdf]

Supplementary Materials

Table S1. Identified metabolites in ^{1}H NMR spectra of aqueous extracts derived from the four groups of skeletal muscle of mice

NO.	Metabolites	δ ^{1}H (ppm) and multiplicity	Moieties
1	Isoleucine	0.93(t), 1.00(d), 1.26(m), 1.42(m), 1.99(m), 3.66(d)	δ-CH_3, γ-CH_3, half γ-CH_2, half γ-CH_2, β-CH, α-CH
2	Leucine	0.94(d), 0.97(d), 1.71(m), 1.74(m), 3.73(m)	α-CH_3, α-CH_3, γ-CH, β-CH_2, α-CH
3	Valine	0.99(d), 1.05(d), 2.26(m), 3.60(d)	γ-CH_3, γ-CH_3, β-CH, α-CH
4	Ethanol	1.17 (t), 3.65 (q)	β-CH_3, CH_2
5	3HB	1.20(d), 2.30(q), 2.39(q), 4.14(m)	γ-CH_3, β-CH_2, γ-CH
6	Alanine	1.47(d), 3.78(q)	β-CH_3, α-CH
7	Acetate	1.91(s)	CH_3
8	Lactate	1.33(d), 4.11(q)	β-CH_3, α-CH
9	Lysine	1.43(m), 1.50(m), 1.73(m), 1.89(m), 1.92(m), 3.02(t), 3.75(t)	γ-CH_2, half γ-CH_2, δ-CH_2, β-CH_2, ε-CH_2, α-CH
10	Glutamate	2.08(m), 2.12(m), 2.34(m), 2.37(m), 3.75(m)	half β-CH_2, half β-CH_2, half γ-CH_2, half γ-CH_2, α-CH
11	Glutamine	2.13(m), 2.42(m), 2.45(m), 3.77(t)	γ-CH_2, β-CH_2, α-CH
12	Glycylproline	4.3(q), 3.9(s), 3.6(m), 2.3(m), 1.9(m)	CH, CH_2, NH_2, N-CH
13	Isocitrate	4.1(d), 3.0(m), 2.6(s), 2.5(s)	CH, CH_2
14	Trimethylamine (TMA)	2.88 (s)	CH_3
15	Creatine	3.04(s), 3.93(s)	N-CH_3, α-CH_2
16	Anserine	7.1(s), 4.5(m), 3.8(s), 3.2(m), 2.7(s)	NH_2, CH_2, CH, CH_3
17	Aspartate	2.68(dd); 2.81(dd); 3.90(dd)	β-CH_2, α-CH
18	Glutathione	2.15(m), 2.55(m), 2.96(m), 3.77(m), 4.56(m)	β-CH_2, γ-CH_2, CH_2-SH, α-CH&CH_2-NH, CH-NH
19	Taurine	3.24(t), 3.41(t)	$^{1}CH_2$, $^{2}CH_2$
20	Glycine	3.57(s)	α-CH_2

21	Glycerol	3.55(dd), 3.64(dd), 3.77(m)	half [1] CH_2, half [3] CH_2, [2] CH
22	Methanol	3.34（s）	CH_3
23	Inosine	3.83(d), 3.84(d), 6.1(d), 8.23(s), 8.35(s)	CH (2), N-CH=N, N-CH'=N
24	AMP	6.14 (d), 8.27 (s), 8.58 (s)	NH_2, δ-CH, [2]CH
25	IMP	4.37(m), 6.14(d), 8.22 (s), 8.58 (s)	CH, NH_2, CH, CH
26	NAD	4.37(m), 4.17(m), 4.69(t), 6.14(d), 8.45(s)	CH, CH, CH, NH_2, CH
27	Glucose	β(3.24 (dd), 3.48 (t), 3.90 (dd)), α(3.54 (dd), 3.71 (t), 3.72 (dd), 3.83 (m))	β(H_2, H_3, H_5), α(H_2, H_3, H_6)
28	UDP-Glucose (UDPG)	5.62 (dd), 6.0 (m)	CH, CH_2
29	Fumarate	6.51(s)	CH
30	Tyrosine	3.05(dd), 3.19(dd), 6.92(d), 7.19(d)	half β-CH_2, half β-CH_2, β-CH, α-CH
31	NADH	4.37(m), 4.17(m), 4.69(t), 6.14(d), 8.45(s)	CH, CH, CH, NH2, CH
32	Histidine	7.06(s), 7.85(s)	[5]CH, [2]CH
33	1-methylhistidine	7.06(s),7.8(s)	CH (2), CH (4)
34	Nicotinurate	8.9(d), 8.7(d), 8.2(t), 7.6(q),	CH, CH_2
35	Phenylalanine	3.12(dd), 3.30(dd), 3.99(dd), 7.33(d), 7.37(t),7.43(t)	α-CH, half β- CH_2, half β-CH_2, α-CH, β-CH, γ-CH
36	Oxypurinol	8.2(s)	N-CH
37	Nicacinamide	7.60(q), 8.23(m), 8.71(m), 8.94(m)	α-CH, β-CH, N-CH, N=CH

Multiplicity: s, singlet; d, double; t, triplet; q, quartet; m, multiple; dd, double of double. Abbreviations: 3HB, 3-hydroxybutyrate; TMA, Trimethylamine; UDPG, UDP-Glucose; IMP, hypoxanthine nucleotide; NADH, Nicotinamide adenine dinucleotide; NAD, nicotinamide adenine dinucleotide; AMP, adenosine monophosphate.

Table S2. Multiple comparisons of relative integration of aqueous metabolites from skeletal muscle of mice.

Metabolites	Mean ± Standard Deviation				Multiple comparisons			One-way ANOVA	
	Con	TMAO	Ex	Ex + TMAO	Ex + TMAO vs. Ex	Ex vs. Con	Ex + TMAO vs. TMAO	F	*P*
Leucine	0.044±0.008	0.045±0.006	0.076±0.017	0.061±0.013	↓	↓↓↓	↑↑	21.191	<0.001
Isoleucine	0.008±0.002	0.009±0.002	0.013±0.004	0.015±0.004	↓↓↓	↑↑↑	↑↑↑	15.544	<0.001
Valine	0.025±0.003	0.019±0.003	0.040±0.011	0.027±0.006	↓↓↓	↑↑↑	↑↑	21.206	<0.001
Ethanol	0.015±0.008	0.034±0.013	0.014±0.008	0.011±0.009	ns	ns	↓↓↓	9.412	<0.001
3-HydroxyButyrate	0.002±0.001	0.002±0.001	0.008±0.003	0.011±0.003	↑↑	↑↑↑	↑↑↑	78.648	<0.001
Alanine	0.224±0.015	0.198±0.024	0.155±0.025	0.130±0.021	ns	↓↓↓	↓↓↓	49.727	<0.001
Lysine	0.027±0.005	0.027±0.005	0.046±0.009	0.035±0.006	↓↓	↑↑↑	↑	22.036	<0.001
Acetate	0.014±0.002	0.018±0.003	0.026±0.003	0.023±0.003	ns	↑↑↑	↑↑	34.294	<0.001
Glycylproline	0.008±0.002	0.005±0.001	0.018±0.005	0.009±0.003	↓↓↓	↑↑↑	↑↑	37.451	<0.001
Glutamate	0.039±0.005	0.028±0.004	0.035±0.012	0.037±0.005	ns	ns	↑	5.402	0.003
Glutamine	0.055±0.005	0.070±0.008	0.028±0.007	0.035±0.005	ns	↓↓↓	↓↓↓	86.803	<0.001
Isocitrate	0.036±0.003	0.044±0.006	0.020±0.004	0.026±0.004	↑	↓↓↓	↓↓↓	66.218	<0.001
Anserine	0.416±0.020	0.472±0.036	0.249±0.061	0.344±0.050	↑↑↑	↓↓↓	↓↓↓	55.104	<0.001
Aspartate	0.001±0.000	0.001±0.000	0.004±0.001	0.003±0.001	↓↓	↑↑↑	↑↑↑	47.488	<0.001
Trimethylamine	0.006±0.002	0.004±0.001	0.001±0.001	0.019±0.015	↑↑↑	ns	↓↓↓	13.639	<0.001
Creatine	1.741±0.096	1.695±0.165	1.474±0.124	1.488±0.147	ns	↓↓↓	↓↓	12.588	<0.001
Taurine	1.724±0.091	1.715±0.165	1.210±0.267	1.493±0.112	↑↑	↓↓↓	↓↓	21.598	<0.001
Methanol	0.426±0.217	0.835±0.260	0.197±0.068	0.304±0.208	ns	ns	↓↓↓	18.085	<0.001
Glycine	0.142±0.011	0.099±0.015	0.078±0.022	0.097±0.012	↑	↓↓↓	ns	35.946	<0.001
Glycerol	0.025±0.004	0.030±0.005	0.017±0.003	0.021±0.003	ns	↓↓↓	↓↓↓	20.697	<0.001
Glutathione	0.208±0.024	0.212±0.027	0.112±0.031	0.145±0.031	↑	↓↓↓	↓↓↓	34.112	<0.001
Lactate	0.747±0.052	0.648±0.099	0.228±0.056	0.316±0.096	ns	↓↓↓	↓↓↓	111.692	<0.001
NAD	0.155±0.006	0.173±0.017	0.128±0.015	0.134±0.012	ns	↓↓↓	↓↓↓	29.212	<0.001

Glucose	0.016±0.002	0.014±0.005	0.001±0.000	0.002±0.002	ns	↓↓↓	↓↓↓	72.559	<0.001
UDPG	0.002±0.000	0.002±0.000	0.002±0.000	0.002±0.000	ns	ns	ns	1.137	0.344
IMP	0.139±0.007	0.141±0.014	0.104±0.017	0.110±0.014	ns	↓↓↓	↓↓↓	25.633	<0.001
Fumarate	0.005±0.001	0.002±0.001	0.006±0.001	0.003±0.001	↓↓↓	↑	↑	32.89	<0.001
NADH	0.074±0.003	0.084±0.007	0.062±0.007	0.065±0.006	ns	↓↓↓	↓↓↓	34.867	<0.001
Histidine	0.062±0.004	0.066±0.006	0.036±0.022	0.049±0.011	ns	↓↓↓	↓↓	14.978	<0.001
Tyrosine	0.006±0.001	0.007±0.001	0.012±0.004	0.008±0.001	↓↓↓	↑↑↑	ns	13.671	0.001
Phenylalanine	0.006±0.001	0.006±0.001	0.008±0.002	0.007±0.001	ns	ns	↑	4.08	0.012
Nicotinurate	0.008±0.000	0.008±0.001	0.007±0.001	0.007±0.001	ns	↓↓	↓↓	9.46	<0.001
1-methylhistidine	0.002±0.001	0.002±0.001	0.001±0.000	0.001±0.000	ns	↓↓↓	↓↓	11.434	<0.001
Oxypurinol	0.001±0.000	0.002±0.000	0.004±0.001	0.004±0.001	ns	↑↑↑	↑↑↑	53.703	<0.001
Inosine	0.005±0.000	0.006±0.001	0.010±0.003	0.009±0.002	ns	↑↑↑	↑↑↑	20.921	<0.001
AMP	0.139±0.008	0.143±0.014	0.104±0.017	0.110±0.014	ns	↓↓↓	↓↓↓	27.028	<0.001
Niacinamide	0.007±0.000	0.007±0.001	0.006±0.001	0.006±0.001	ns	↓↓	↓↓↓	10.443	<0.001

* Note: ↓: downregulated; ↑: upregulated. ns, P > 0.05; ↓/↑, P < 0.05; ↓↓/↑↑, P < 0.01; ↓↓↓/↑↑↑, P < 0.001; ↓↓↓↓/↑↑↑↑, P < 0.0001. Abbreviations: UDPG, UDP-Glucose; IMP, hypoxanthine nucleotide; NADH, Nicotinamide adenine dinucleotide; NAD, nicotinamide adenine dinucleotide; AMP, adenosine monophosphate.

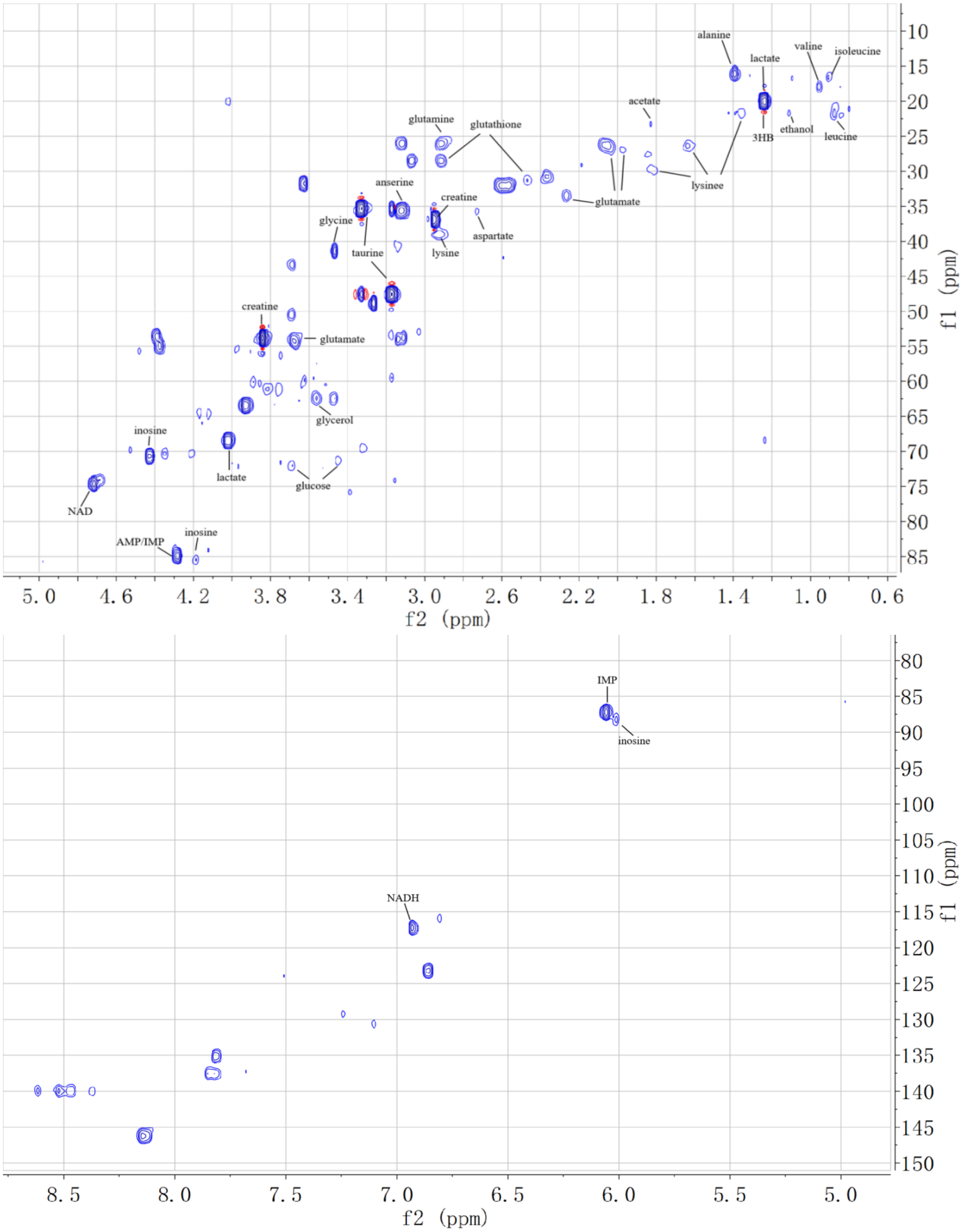

Figure S1. Typical 2D ^{1}H-^{13}C HSQC spectrum of aqueous extracts derived from the four groups of mouse skeletal muscles recorded on 850 MHz NMR spectrometer.

Table S3. Metabolic pathways were significantly altered in skeletal muscles of four groups of mice.

**Metabolic pathways**	**Ex+TMAO vs.Ex**	**Ex vs.Con**	**Ex+TMAO vs.TMAO**
alanine, aspartate and glutamate metabolism		√	√
D-Glutamine and D-glutamate metabolism		√	√
taurine and hypotaurine metabolism		√	√
glutathione metabolism	√	√	√
histidine metabolism	√	√	√
nicotinate and nicotinamide metabolism		√	√
glycine, serine and threonine metabolism		√	
phenylalanine, tyrosine and tryptophan biosynthesis	√	√	√
phenylalanine metabolism		√	√
glycerolipid metabolism		√	√

Table S4. The design of 2-week adaptive swimming training

Date		training time (min)	Weight (%)*
Week 1	Day 1	10	0
	Day 2	20	0
	Day 3	30	0
	Day 4	40	0
	Day 5	50	0
Week 2	Day 1	30	1
	Day 2	30	2
	Day 3	30	3
	Day 4	30	4
	Day 5	30	5

Note: * The loading weight of mouse is the percentage of its body weight.
